# Supplementary figures and images for: The Zymoseptoria tritici effector Zt-11 contributes to aggressiveness in wheat
Source: PLoS One. 2024 Nov 19;19(11):e0313859. doi: 10.1371/journal.pone.0313859 (PMC11575801; doi:10.1371/journal.pone.0313859)

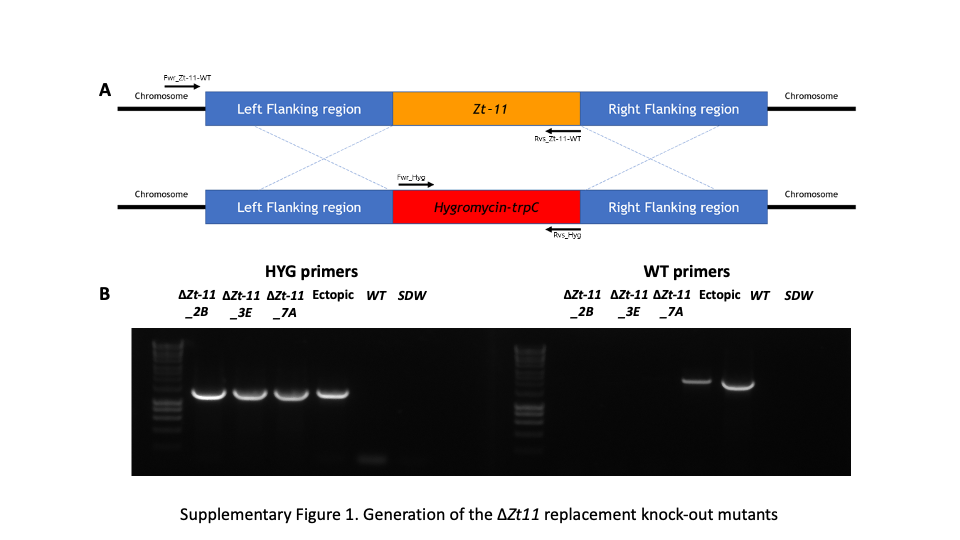

Supplement: S1 Fig — A, Diagram showing the location of the two primer pairs used to confirm successful transformation of Z. tritici. The replacement of Zt11 by the hygromycin-trpC resistance cassette through homologous recombination is depicted by dotted blue lines on the flanking regions. B, Successful disruption of the Zt11 gene in the mutants indicated by presence of the KO amplicon (2340 base pairs) and absence of the Zt11 wild-type WT amplicon (2124 base pairs). 1kb (kilobase) ladder (Bioline). (TIF) [file pone.0313859.s001.tif]

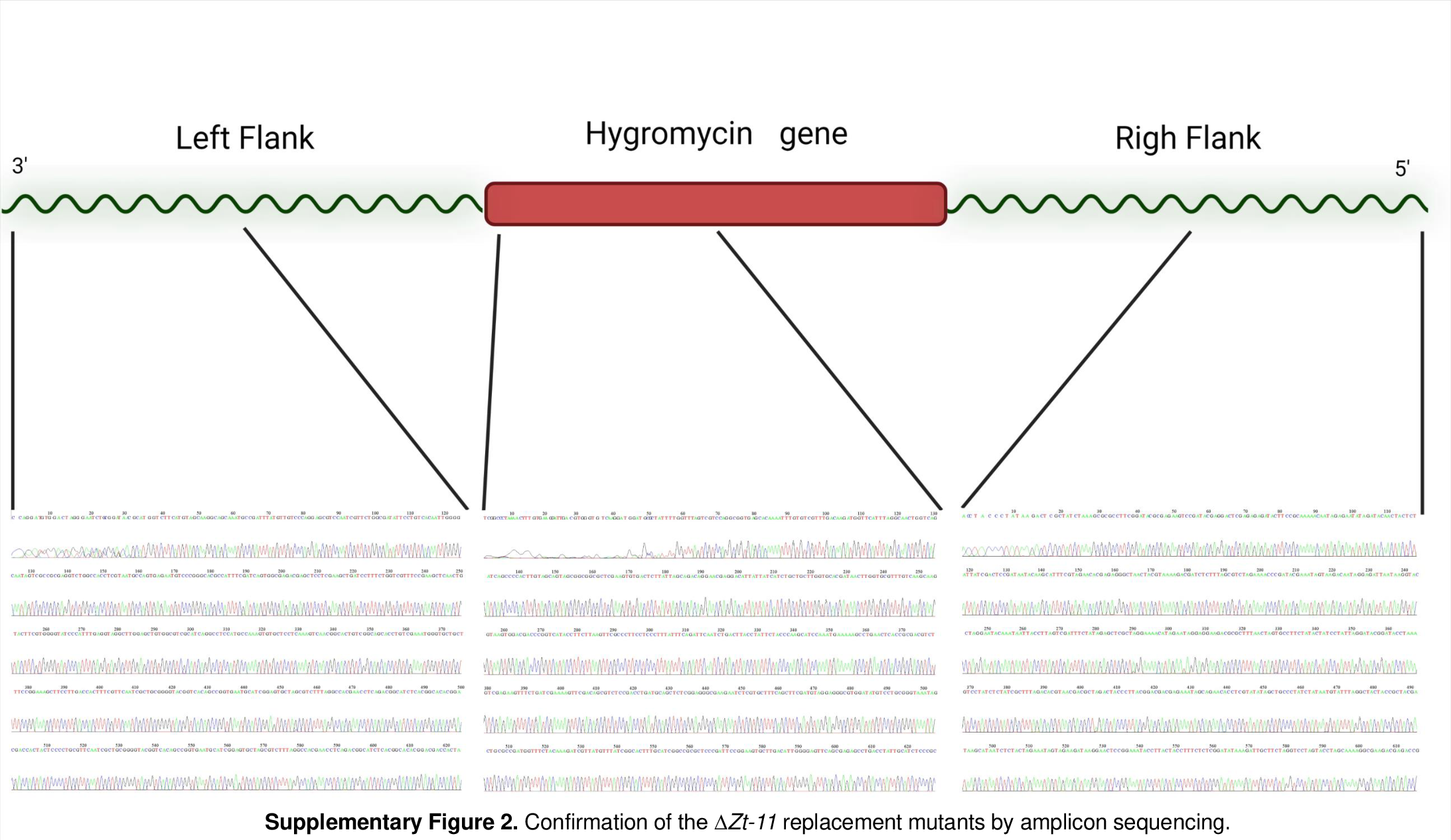

Supplement: S2 Fig — Each row represents sequences (left flank, hygromycin and right flank) confirmed by amplicon sequencing in the mutant strains. (TIF) [file pone.0313859.s002.tif]

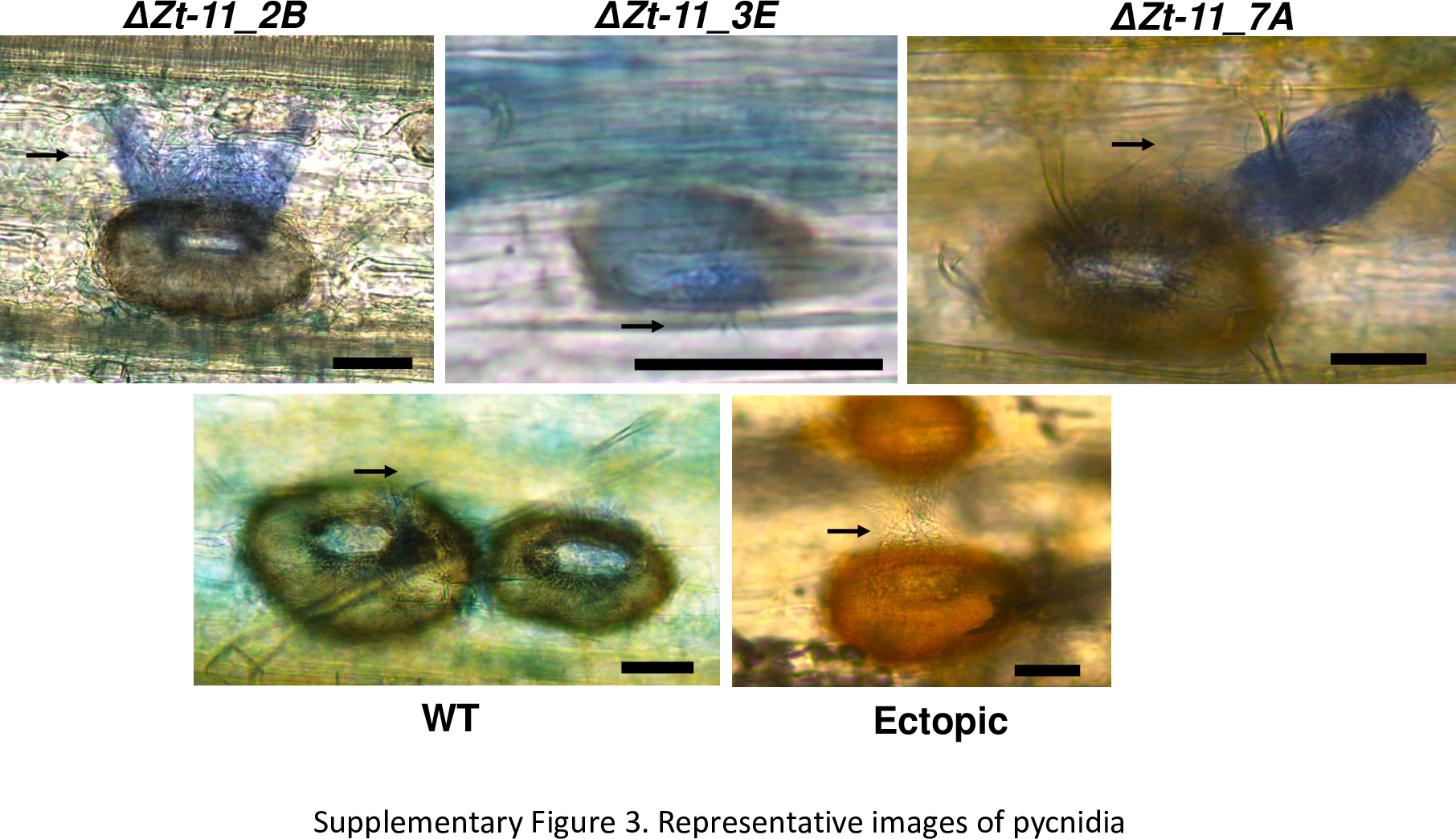

Supplement: S3 Fig — ΔZt11 mutant strain, WT (IPO323) and ectopic strain pycnidia on wheat cv. Longbow leaves at 21 dpi. Arrows indicate the cirrhus containing pycnidiospores, scale bars representative of 50μm. (TIF) [file pone.0313859.s003.tif]

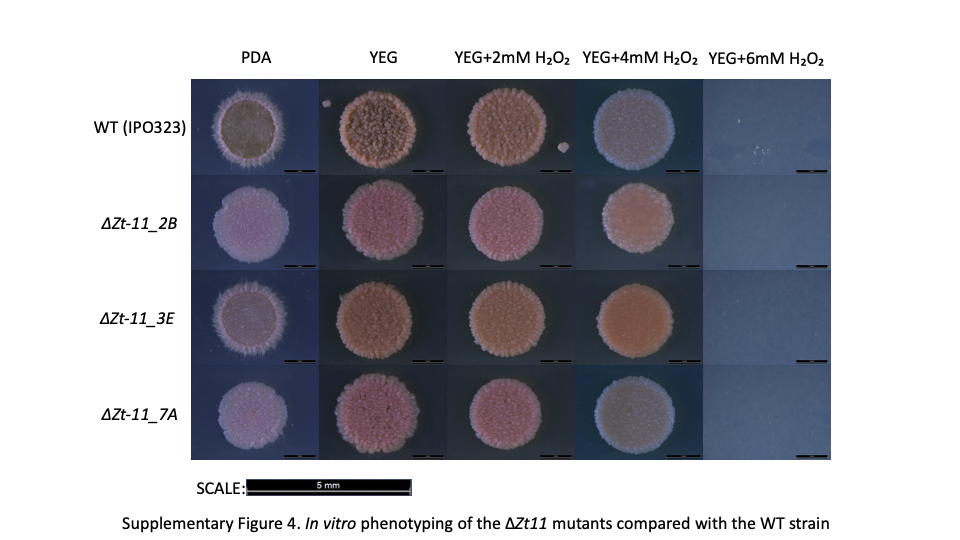

Supplement: S4 Fig — Approximately 107 spores/mL of spores was spotted on to three different solid growth media; PDA, YPDA and YEG (supplemented with H2O2 at concentrations 0, 2, 4 and 6mM) and incubated for 7 days at 20°C. Scale bar = 5mm. Images are representative of three independent experiments, with a total of 2 plates per experiment per media type (n = 4). (TIF) [file pone.0313859.s004.tif]

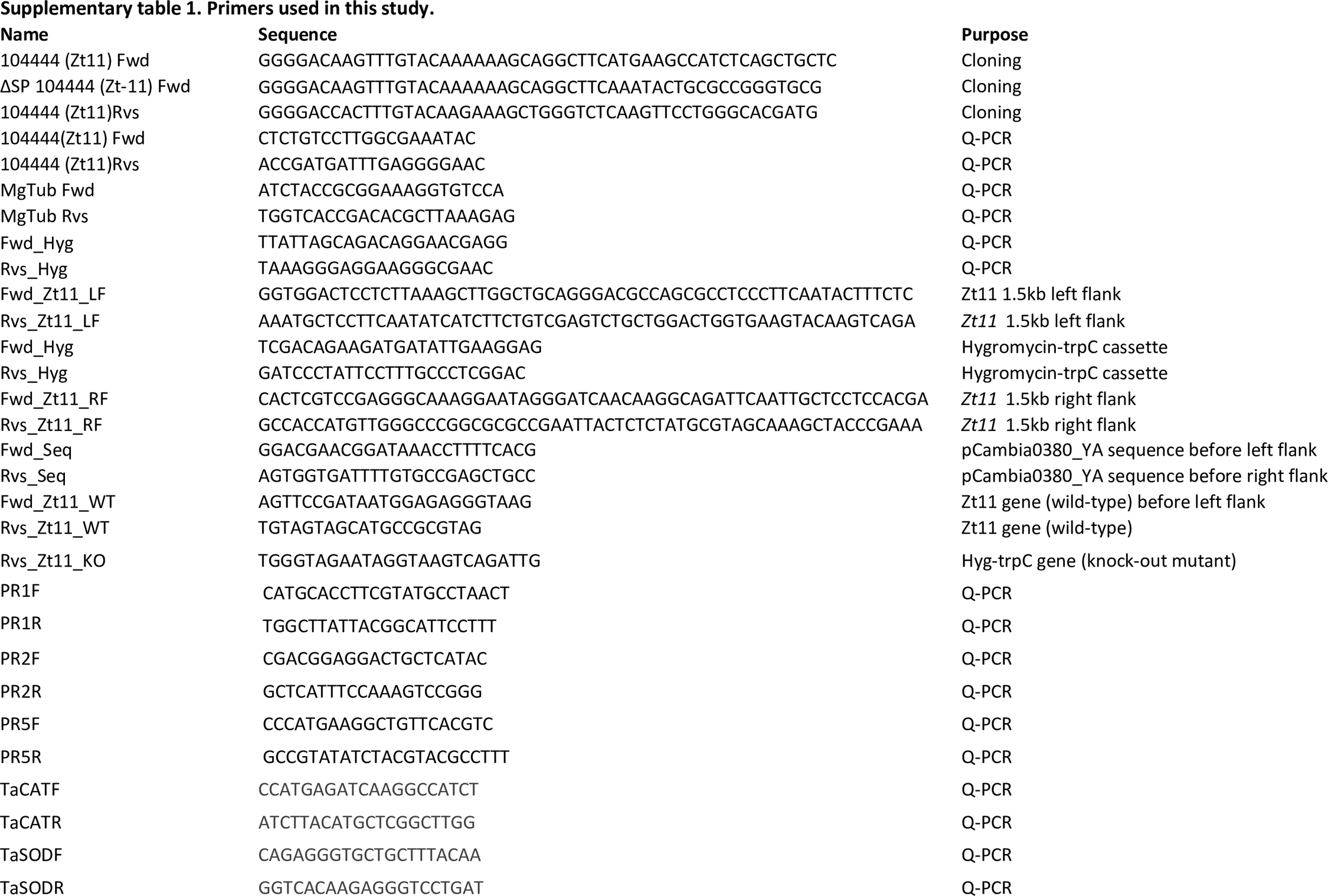

Supplement: S1 Table — (TIF) [file pone.0313859.s005.tif]

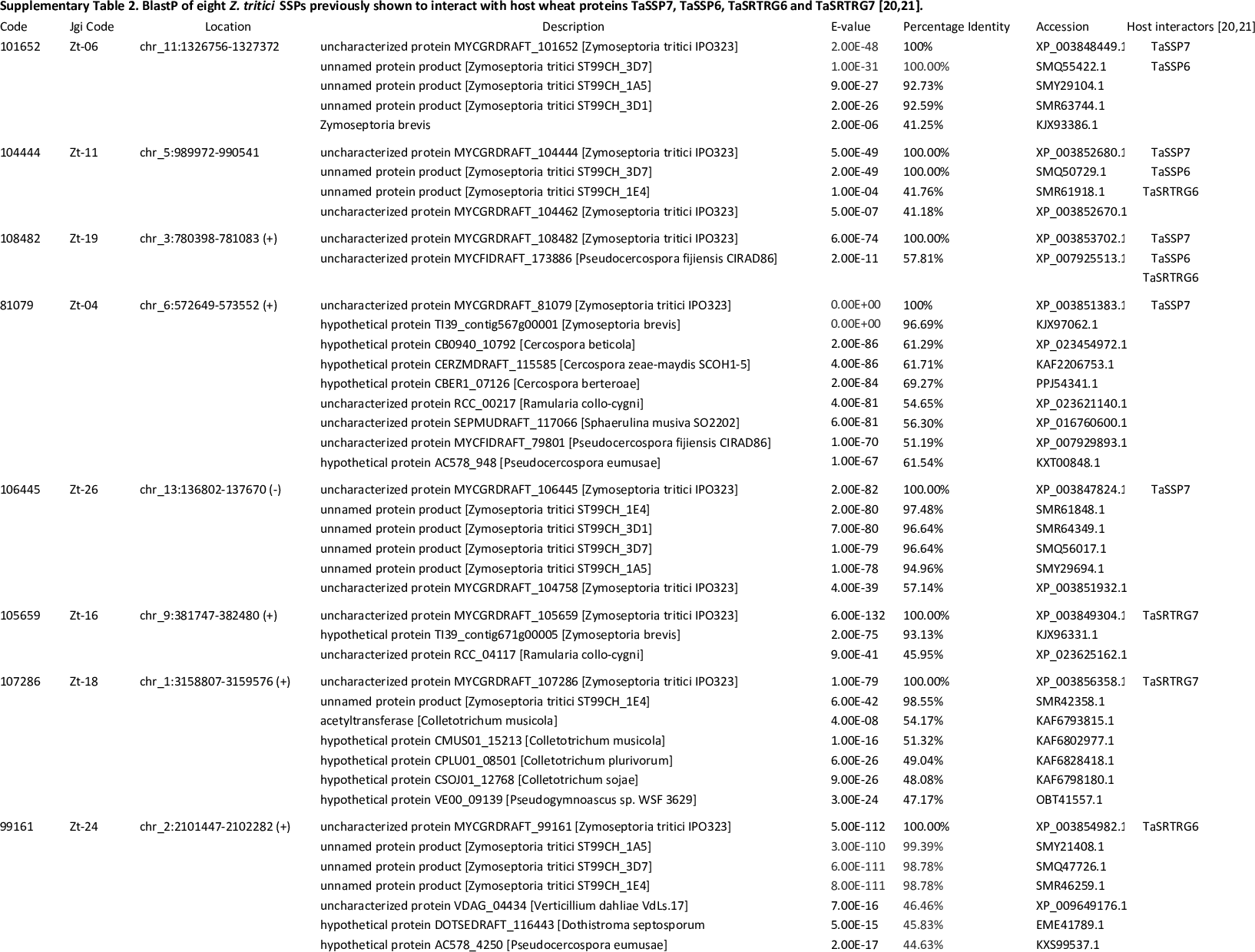

Supplement: S2 Table — (TIF) [file pone.0313859.s006.tif]
